# Evaluation of the Antibacterial Potential of Liquid and Vapor Phase Phenolic Essential Oil Compounds against Oral Microorganisms

**DOI:** 10.1371/journal.pone.0163147

**Published:** 2016-09-28

**Authors:** Tong-Hong Wang, Shih-Min Hsia, Chi-Hao Wu, Shun-Yao Ko, Michael Yuanchien Chen, Yin-Hua Shih, Tzong-Ming Shieh, Li-Chuan Chuang, Ching-Yi Wu

**Affiliations:** 1 Tissue Bank, Chang Gung Memorial Hospital, Tao-Yuan, Taiwan; 2 Research Center for Industry of Human Ecology, Chang Gung University of Science and Technology, Tao-Yuan, Taiwan; 3 Graduate Institute of Health Industry Technology, Chang Gung University of Science and Technology, Tao-Yuan, Taiwan; 4 School of Nutrition and Health Sciences, Taipei Medical University, Taipei, Taiwan; 5 Graduate Institute of Medical Science, College of Health Science, Chang Jung Christian University, Tainan, Taiwan; 6 Innovate Research Center of Medicine, Chang Jung Christian University, Tainan, Taiwan; 7 Department of Oral & Maxillofacial Surgery, China Medical University Hospital, Taichung, Taiwan; 8 School of Dentistry, College of Medicine, China Medical University, Taichung,Taiwan; 9 Mind-Body Interface Lab, China Medical University Hospital, Taichung, Taiwan; 10 Department of Dental Hygiene, College of Health Care, China Medical University, Taichung, Taiwan; 11 Department of Pediatric Dentistry, Chang Gung Memorial Hospital at Linkou, Taoyuan, Taiwan; 12 Graduate Institute of Craniofacial and Dental Science, College of Medicine, Chang Gung University, Taoyuan, Taiwan; 13 Institute of Oral Biology, National Yang-Ming University, Taipei, Taiwan; Jamia Millia Islamia, INDIA

## Abstract

The aim of the present study was to determine the antibacterial activities of the phenolic essential oil (EO) compounds hinokitiol, carvacrol, thymol, and menthol against oral pathogens. *Aggregatibacter actinomycetemcomitans*, *Streptococcus mutans*, Methicillin-resistant *Staphylococcus aureus* (MRSA), and *Escherichia*. *coli* were used in this study. The minimum inhibitory concentrations (MICs), minimum bactericidal concentrations (MBCs), bacterial growth curves, temperature and pH stabilities, and synergistic effects of the liquid and vapor EO compounds were tested. The MIC/MBC of the EO compounds, ranging from the strongest to weakest, were hinokitiol (40–60 μg/mL/40-100 μg/mL), thymol (100–200 μg/mL/200-400 μg/mL), carvacrol (200–400 μg/mL/200-600 μg/mL), and menthol (500-more than 2500 μg/mL/1000-more than 2500 μg/mL). The antibacterial activities of the four EO phenolic compound based on the agar diffusion test and bacterial growth curves showed that the four EO phenolic compounds were stable under different temperatures for 24 h, but the thymol activity decreased when the temperature was higher than 80°C. The combination of liquid carvacrol with thymol did not show any synergistic effects. The activities of the vaporous carvacrol and thymol were inhibited by the presence of water. Continual violent shaking during culture enhanced the activity of menthol. Both liquid and vaporous hinokitiol were stable at different temperatures and pH conditions. The combination of vaporous hinokitiol with zinc oxide did not show synergistic effects. These results showed that the liquid and vapor phases of hinokitiol have strong anti-oral bacteria abilities. Hinokitiol has the potential to be applied in oral health care products, dental materials, and infection controls to exert antimicrobial activity.

## Introduction

Essential oils (EOs) are volatile oily liquids obtained from different parts of plants. EOs are widely used in food preservation and health care products because of their potent antibacterial activity [1–3], reduction of oxidative stress [4], and anti-inflammatory activities [5]. Many EOs are generally recognized as safe by the Food and Drug Administration (FDA) of the United States and have been used as artificial flavorings and preservatives. EOs are often diluted in solvents for sprays and rinses or are heated to volatilize them to prohibit bacterial growth and eliminate unpleasant odors. Many EOs contain terpenoids, which include phenols, aldehydes, ketones, alcohols, ethers, and hydrocarbons. Generally, phenolic EOs have stronger antibacterial activity than other constituents. The antibacterial activities of the terpenoids are affected by their functional groups, hydrophobicity, and environmental conditions.

The antibacterial activity of the constituents in EOs against cariogenic bacteria has been extensively discussed [6,7]. Hinokitiol is a natural component isolated from *Chamacyparis taiwanensis*. It has already been demonstrated that an oral care gel (therapeutic dentifrice) containing hinokitiol improved the quality of life for oral lichen planus patients [8] and effectively for reduced oral malodor [9]. The EO of *Lippia gracilis Schauer* leaves has significant synergism with several antibiotics [10]. The bioactive fractions of *Lippia sidoides* disrupt the integrity and weaken the structure of biofilms [11]. Using *L*. *sidoides*-based essential oil mouth rinse for one week was efficacious in reducing bacterial plaques and gingival inflammation in patients [12], and it reduced the salivary *Streptococcus mutans* levels in children with caries after five days of treatment [13].

The major constituents of *L*. *gracilis* and *L*. *sidoides* are carvacrol and thymol [10,14]. Carvacrol and thymol have been used as food additives because of their antimicrobial and antioxidant activities [15,16]. Thymol can also be used in varnish to prevent caries [17], and carvacrol has well-known anti-*Candida* potential and can prevent denture stomatitis [14]. Menthol is either made synthetically or obtained from mint. Menthol is used in confections, chewing gum, and oral-care products, such as toothpaste and mouth rinse, to reduce bacterial growth [18] and oral malodor [19]. These four phenolic EO compounds are valuable for application as food additives or oral health care products.

Dental caries and periodontitis represent the major oral infectious diseases. Bacterial plaques composed of native oral flora accumulate on dental surfaces and are the primary etiological agents of periodontal disease and dental caries [20]. In dental plaques, *S*. *mutans* and *Aggregatibacter actinomycetemcomitans* are respectively considered to be highly cariogenic and periodontopathic microorganisms. Staphylococcal food poisoning is caused by consuming foods contaminated with enterotoxins produced by *Staphylococcus aureus* [21]. Methicillin-resistant *Staphylococcus aureus* (MRSA) are facultative-anaerobic *Staphylococci*, and they have been reported to colonize 77.8% of oral cancer patients following surgery [22]. *Escherichia*. *coli* can cause serious food poisoning in humans. Fecal-oral transmission is the major route by which *E*. *coli* is transmitted to induce enteric diseases. *E*. *coli* has been used as an ideal indicator organism to test environmental samples for fecal contamination.

**Fig 1:**
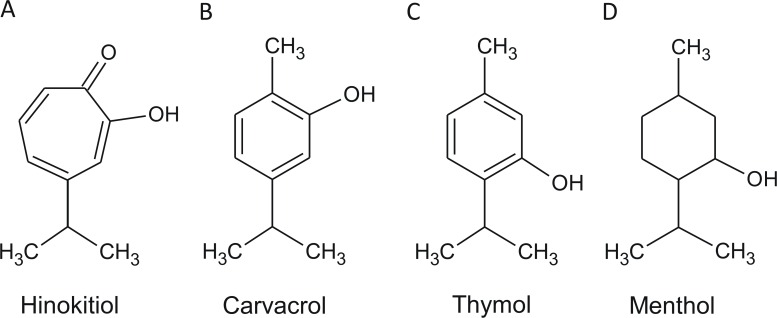
The chemical structures of the phenolic EO compounds. (A) hinokitiol; (B) carvacrol; (C) thymol; (D) menthol.

Hinokitiol, carvacrol, thymol, and menthol have similar structures and molecular weights (Fig 1). Carvacrol and thymol are structural isomers but have distinct physical characteristics. Carvacrol is a liquid at room temperature because it has a low melting point, while the others are powders at room temperature. Menthol melts near human body temperature, and hinokitiol and thymol both melt at 50°C. The vapor pressure of hinokitiol is lower than that of the other compounds. Carvacrol, thymol, and menthol tend to evaporate or volatilize easily at moderate temperatures, while hinokitiol does not (Table 1). These phenolic EO compounds are used in combination with other materials at different concentrations, pH, and temperatures in various health care products. The antibacterial activity of hinokitiol is synergistically increased when combined with zinc oxide, and the combination of carvacrol with thymol was also shown to have synergistic effects [23,24].

**Table 1:** The physical characteristics of hinokitiol, carvacrol, thymol, and menthol.

	Hinokitiol	Carvacrol	Thymol	Menthol
**Molecular weight**	164.2	150.22	150.22	156.27
**Formula**	C_10_H_12_O_2_	C_10_H_14_O	C_10_H_14_O	C_10_H_20_O
**Density, 25°C (g/cm^3^)**	1.127	0,977	0,965	0,89
**Vapor pressure, 25°C (mm/Hg)**	8.9×10^−5^	2.96×10^−2^	3.76×10^−2^	3.20×10^−2^
**Boiling point, 1 atm (°C)**	303.4	236 ~ 237	231~ 232	214 ~ 216
**Melting point, 1 atm (°C)**	48~53	3~4	49~51	34~36

Fully understanding the antibacterial activities of these four phenolic EO compounds in different states and under different conditions would be helpful for choosing suitable additives for various health care products. In this study, *A*. *actinomycetemcomitans*, *S*. *mutans*, MRSA, and *E*. *coli* were used as disease indicators for periodontal disease, caries, infection, and enteric diseases, respectively, to test the antibacterial potential of hinokitiol, carvacrol, thymol, and menthol in the liquid and vapor phases under various temperature and pH conditions and at different mix ratios. The results of these studies provide information that can help to generate effective new applications for novel dental formulations, food additives, oral health foods, and infection control.

## Materials and Methods

### Antimicrobial agents and chemicals

Hinokitiol (469521), carvacrol (282197), thymol (T0501), menthol (M2772), zinc oxide (ZnO, 721077), and chlorhexidine (CHX, 282227) were purchased from Sigma-Aldrich (St. Louis, MO, USA). The EOs were dissolved or diluted in DMSO, and ZnO was dissolved in 2.5 N HCl. All of the compounds were made as stock solutions of 100 mg/mL and were stored at -20°C. The chemical structures and physical characteristics of these four phenolic EO compounds are shown in Fig 1 and Table 1.

### Microorganisms and media

*A*. *actinomycetemcomitans* (ATCC number: 33384), *S*. *mutans* (ATCC number: 25175), Methicillin-resistant *S*. *aureus* (MRSA, ATCC number: 33591), and *E*. *coli* (ATCC number: 10798) were used in the study. *A*. *actinomycetemcomitans* was cultured in brain heart infusion (BHI) broth, *S*. *mutans* and MRSA were cultured in tryptic soy broth (TSB), and *E*. *coli* was cultured in Lysogeny broth (LB). The bacteria were inoculated by loop transfer from frozen tubes into 3 mL slant nutrient broth, then were subjected to 200 rpm shaking culture at 37°C for 24 h. Bacteria from these cultures were transferred onto an appropriate solid medium and incubated overnight. Selected colonies were transferred to the appropriate liquid medium and were incubated for 4–6 h to achieve log phase growth. The optical density of each culture at 600 nm (OD600) was adjusted to 1.0 using fresh broth to give a standard inoculum of 10^6^ cfu/mL. Stock cultures were maintained at -80°C in growth broth containing 25% sterile glycerol.

### Direct contact agar diffusion tests

For direct contact agar diffusion tests, 5 mL of fresh broth agar was prepared in 6-cm Petri dishes, and bacteria were spread at 5×10^5^ cfu on the broth agar surface. Aliquots (4–10 μL) of the different test compounds (200 μg-1000 μg) were placed on 6-mm diameter filter discs. Using the direct contact method, the discs were placed on the center of the solidified agar surface. The cultures were incubated for 24–96 h at 37°C, and the diameter of the inhibition zone was then recorded.

### Minimum inhibitory concentration (MIC) and minimum bactericidal concentration (MBC) of phenolic EO compounds determined by the broth dilution method

Cell suspensions were prepared in 2 mL of broth with various concentrations of the phenolic EO compounds in 15 mL culture tubes by inoculation with 2 μl of 10^6^ cfu/mL from each glycerol stock. The cultures were incubated at 37°C at 200 rpm for 24 h. Tubes showing no visible turbidity were considered to represent the MIC and were subsequently inoculated onto sterile 6 cm nutrient agar plates without any phenolic EO compound and incubated for 24 h. The lowest concentration at which no growth was observed was considered to be the MBC [23].

### Growth curve assay

The growth curve assay was conducted in a 96-well format that was adapted from a previously described method [25]. Bacterial suspensions prepared with various concentrations of phenolic EO compounds in 1 mL of liquid broth in 1.6 mL microcentrifuge tubes were inoculated with 1 μL of 10^6^ cfu/mL from the glycerol stocks, 200 μL were then transferred to 96-well plates for testing, and 200 μL of sterile liquid broth was used as a blank. The 24-h growth curve analyses were performed for the four oral pathogens at 37°C. The kinetic analysis included a 10-s shaking step before each of the time point measurements of the OD600, which were recorded at 30 min intervals. The data were analyzed using the VersaMax^TM^ and Softmax® Pro (version 5.4.1, California, US) software programs.

### Heat stability test

To evaluate the stabilities of the phenolic EO compounds at different temperatures, the test compounds were pre-incubated at 4°C, 25°C, 50°C, 80°C, and 100°C for 1 h for a heat stability test, followed by direct contact diffusion tests. The diameter of the inhibition zone was recorded.

### Vapor phase agar diffusion tests

The agar diffusion test was used to evaluate the antibacterial activities of the phenolic EO compounds in the vapor phase, and it was technically similar to the direct contact diffusion test, with the same 6 cm Petri dish format, bacterial culture, filter disc size, and EO compound loading [26]. However, the filter discs were placed in the center of the cover of the Petri dish in this experiment. The dishes were then sealed using laboratory parafilm to avoid evaporation of the test compounds, followed by incubation at 37°C for 24–96 h. The diameter of the inhibition zone was recorded.

### Stability of the phenolic EO compounds under various pH conditions

The pH of the water was adjusted to pH 3, pH 5, pH 7, pH 9, and pH 11 by adding HCl or NaOH, and it was measured by a pH meter before use. A total of 500 μg of each phenolic EO compound was dissolved in 5 μL DMSO, which was then mixed with 5 μL of water with different pH values (pH 3 to pH 11). Then, the vapor phase agar diffusion test was performed. The diameter of the diffusion zone was recorded.

### Statistics

All of the assays were performed in duplicate or triplicate. Differences between specific means were analyzed by a one-way analysis of variance (ANOVA). Group means were compared using a one-way ANOVA and Tukey’s test. The data are shown as the means ± standard deviation (SD). Differences between the variants were considered significant when *P* < 0.05. The CompuSyn software (Version 1.0, ComboSyn Inc., USA) was used to quantify synergism and antagonism for the drug combinations. All the raw data was showed in S1 File.

## Results

### Antibacterial activity of the four phenolic EO compounds

**Fig 2:**
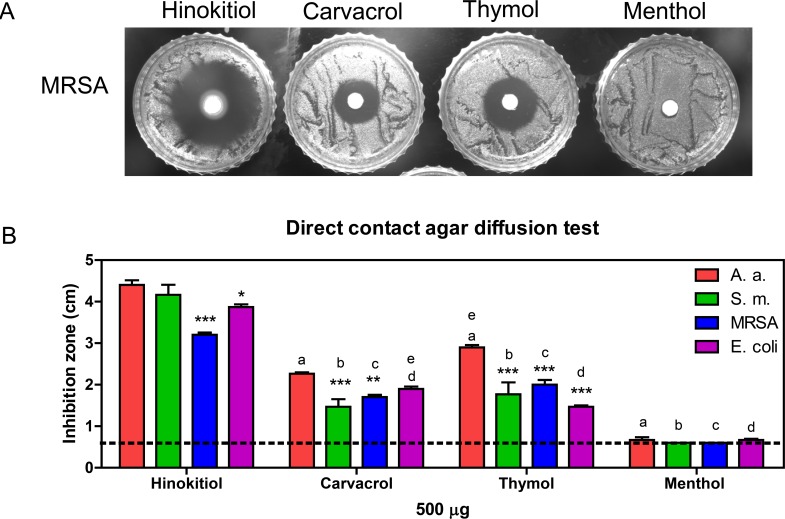
The antibacterial activities of the phenolic EO compounds. (A) MRSA treated with 500 μg phenolic EO compounds, as assessed using direct contact agar diffusion tests. (B) The phenolic EO compounds were all tested at 500 μg. The microorganisms examined were *A*. *actinomycetemcomitans* (Aa), *S*. *mutans* (Sm), MRSA, and *E*. *coli*. Dotted line, the 0.6 cm diameter of the filter disc. * *P* < 0.05, ** *P* < 0.01, *** *P* < 0.001 compared with *A*. *a*. in each compound group; a, b, c, and d were *P* < 0.05, compare with *A*. *a*., *S*. *m*., MRSA, and *E*. *coli* in the hinokitiol group, respectively; e, *P* < 0.01 based on a comparison of the carvacrol and thymol groups.

All of the test compounds were used at 500 μg in the direct contact diffusion tests. Hinokitiol showed the largest inhibition zone, and menthol showed little inhibition in this study. Although carvacrol and thymol are structural isomers, they showed different inhibition zones for all of the bacteria tested. Fig 2A shows the results of the direct contact agar diffusion test of the four phenolic EO compounds against MRSA. *A*. *actinomycetemcomitans* was more sensitive to the phenolic EO compounds than the other bacteria. The inhibition zones for *A*. *actinomycetemcomitans*, *S*. *mutans*, and MRSA were the largest for hinokitiol, followed by thymol, carvacrol, then menthol. However, *E*. *coli* was more sensitive to carvacrol than thymol. The diameter of the inhibition zone for menthol was 0.667 ± 0.116 cm in *A*. *actinomycetemcomitans* and 0.667 ± 0.058 cm in *E*. *coli*, but there was no inhibition zone in the dishes with *S*. *mutans* and MRSA (Fig 2B). The diameter of the inhibition zone in our analysis is shown by the solid column/symbol and hollow column/symbol representing the direct contact and vapor phase agar diffusion method, respectively. The dotted line represents the 0.6 cm diameter of the filter disc used in the direct contact agar diffusion method, while this was not used in the vapor phase agar diffusion method. Because the diameters of the inhibition zones were totally formed by the gaseous phenolic compounds in the vapor phase studies, we did not include the filter disc coverage for those samples.

### The MIC and MBC of the four phenolic EO compounds

Different concentration ranges of the four phenolic EO compounds were tested by the broth dilution method to determine the MIC and MBC. Hinokitiol was tested from 20 to 120 μg/mL, carvacrol and thymol from 50 to 1000 μg/mL, and menthol from 250 to 2500 μg/mL. Chlorhexidine (CHX) is commonly used as an active ingredient in mouth rinse to reduce dental plaques and oral bacteria. Hence, CHX was used as a positive control and was tested at concentrations ranging from 0.5 to 4 μg/mL. The MIC and MBC of the test phenolic EO compounds against the four oral pathogens are listed in Table 2. The results of the inhibition zone (Fig 2B) and MIC/ MBC (Table 2) experiments were consistent. Hinokitiol was a strong antiseptic, carvacrol and thymol were relatively moderate antiseptics, and menthol was a weak antiseptic.

**Table 2:** The MIC and MBC of the four phenolic EO compounds against four microorganisms (μg/mL).

	Aa		Sm		MRSA		*E*. *coli*	
	MIC	MBC	MIC	MBC	MIC	MBC	MIC	MBC
**Hinokitiol**	40	40	40	100	60	60	40	100
**Carvacrol**	200	200	400	600	400	600	400	400
**Thymol**	100	200	200	400	200	200	200	400
**Menthol**	500	1000	1000	1000	1000	1000	>2500	>2500
**CHX**	1	1	1	1	1	2	1	1

MIC and MBC data for phenolic EO compounds and chlorhexidine (CHX; positive control) in *A*. *actinomycetemcomitans* (Aa), *S*. *mutans* (Sm), MRSA, and *E*. *coli* as determined in three independent experiments using the broth dilution method.

### Microorganism growth is delayed in a concentration-dependent manner by the four phenolic EO compounds

**Fig 3:**
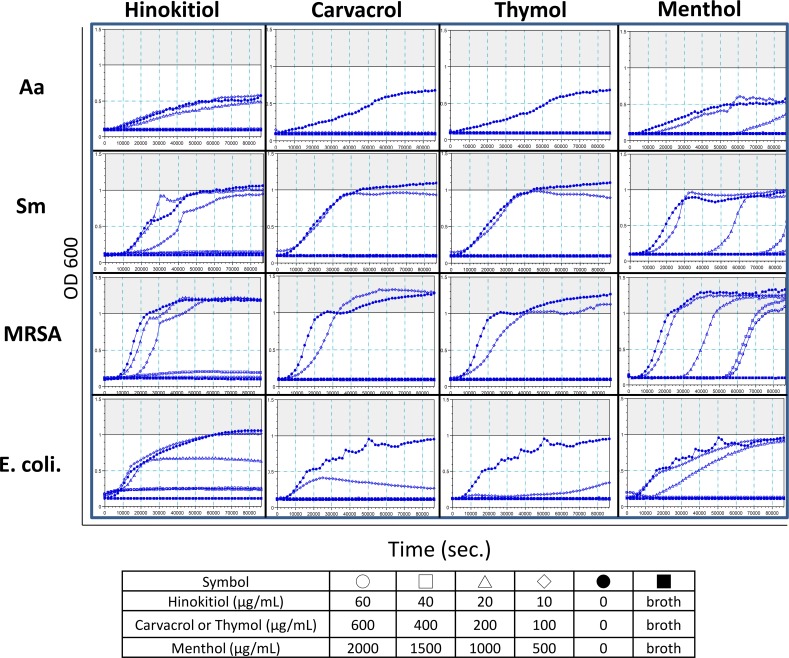
Phenolic EO compounds delay the microorganism growth curves in a concentration-dependent manner. Various concentrations of the phenolic EO compounds were used to test their impact on the bacterial growth curves. The bacterial growth curves in the presence of various phenolic EO concentrations (hollow diamond, triangle, square, and circle) were compared to each control (solid circle). Broth-only treatment served as a negative control (solid square). Y axis, OD600; X axis, time (sec).

The kinetic microplate method was used to analyze the bacterial growth inhibition for 24 h. A log phase delay or a delay in the stationary phase of the growth curve after a 24-h incubation implies that bacterial growth was inhibited or that the phenolic EO compounds killed the bacteria, respectively. Interestingly, the cultures with a delay in the stationary phase (based on the OD600 of bacterial cultures) were more common in the samples treated with low concentrations of phenolic EO compounds than in the control samples, which might have been caused by bacterial aggregation in the culture, such as in MRSA cultures treated with 100 μg/mL carvacrol. The results for the hinokitiol group were consistent with the MIC of each microorganism examined in the study. In the carvacrol and thymol groups, the concentrations that affected the microorganisms’ growth curves (less than 100–200 μg/mL) were lower than the MIC (100–400 μg/mL), but this finding was inverted in the menthol group (Fig 3). Overall, the log phases of the microorganisms’ growth curves were dose-dependently delayed, except for the *S*. *mutans* groups treated with 10 and 20 μg/mL hinokitiol.

### The phenolic EO compounds are heat stable

**Fig 4:**
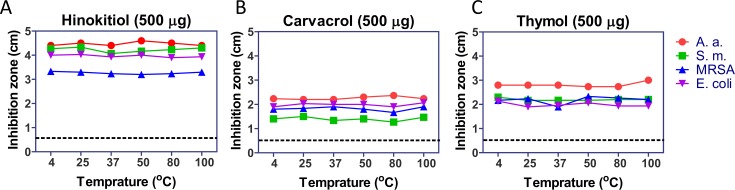
The phenolic EO compounds were heat stable. The phenolic EO compounds (500 μg) were pre-incubated at 4–100°C for 1 h before the direct contact agar diffusion test. (A) Hinokitiol; (B) carvacrol; (C) thymol. Dotted line, the 0.6 cm diameter of the filter disc.

After 500 μg of hinokitiol, carvacrol, and thymol were pre-incubated at different temperatures (4 to 100°C) for 1 h, the inhibition zones were not significantly different for the four oral pathogens based on the direct contact agar diffusion test (Fig 4). The antibacterial activities of the heated phenolic EO compounds from strongest to weakest were consistent with previous findings for the compounds (Fig 2B, Table 2). However, when two to three EO-loaded discs were placed in a 10-cm dish to perform direct contact diffusion tests, the bacterial colony number and size were decreased, and the inhibition zones increased. The phenomenon was not observed in the CHX group (data not shown). These results suggested that the EO phenolic compounds might evaporate to interfere with bacterial growth, and the molecular diffusion could be excluded as a factor affecting the findings. The inhibition zones of 500 μg menthol were excluded due to its weak antibacterial activity.

### The vapor phenolic EO compounds display antibacterial activity

**Fig 5:**
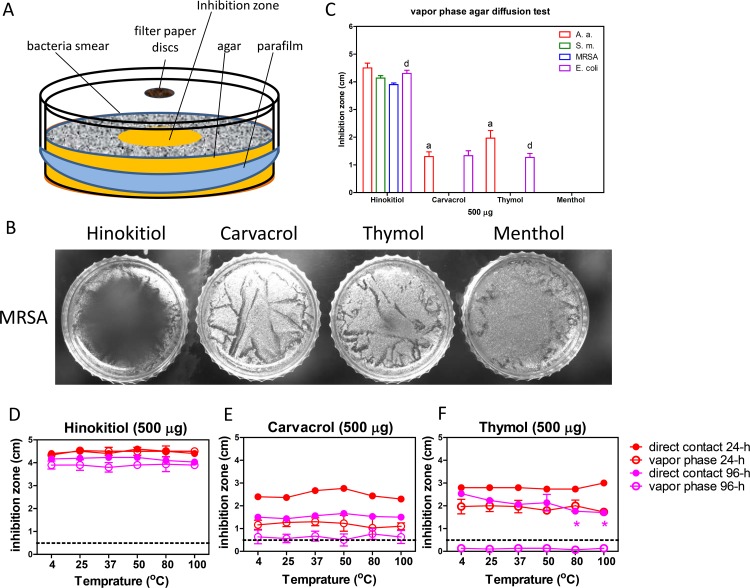
The vaporous phenolic EO compounds display antibacterial activity. (A) The vapor phase agar diffusion experimental device. (B) MRSA treated with 500 μg phenolic EO compounds was examined by vapor phase agar diffusion tests. (C) The vapors from 500 μg phenolic EO compounds were tested by vapor phase agar diffusion. The liquid and vapor phases of (D) hinokitiol, (E) carvacrol, and (F) thymol showed different antibacterial activities after incubation at different temperatures. *P < 0.05, compared with 4°C in each curve. Dotted line, the 0.6 cm diameter of the filter disc.

To verify the antibacterial activity of the phenolic EO compounds due to evaporation at 37°C, the vapor phase agar diffusion test was performed (Fig 5A). Vaporous hinokitiol also showed the best antibacterial activity out of the four compounds tested in the study. Vaporous carvacrol and thymol showed small and clear inhibition zones in Gram-negative bacteria (*A*. *actinomycetemcomitans* and *E*. *coli*) but weak activity against Gram-positive bacteria (*S*. *mutans* and MRSA). The *S*. *mutans* and MRSA colonies were small and thin, meaning that there was weak inhibition by volatile carvacrol and thymol. The vaporous menthol did not show any inhibition zone (Fig 5B). However, the indistinct margin of inhibition zone measurements may have led to some error in determining the sizes of the inhibition zones (Fig 5C).

We used *A*. *actinomycetemcomitans* to compare the antibacterial activities of liquid and vapor phenolic EO compounds pre-incubated at various temperatures by direct contact and evaporation conditions, respectively. The inhibition zones of hinokitiol were not significantly different between the direct contact and vapor phases after 24 h. The inhibition zones of both direct contact and the vapor phase for hinokitiol were reduced after 96 h, and the inhibition zones of the vapor phase were smaller than those in the direct contact group. The liquid and vapor forms of hinokitiol were stable when subjected to freezing, refrigeration, room temperature, and high temperature, and the antibacterial activity of this EO was not significantly different for the different forms or after storage at different temperatures (Fig 5D). The inhibition zones of vapor carvacrol and thymol were smaller than those obtained by the direct contact method at both 24 and 96 h. Although, the antibacterial activity of thymol was stronger than that of carvacrol (Figs 2 and 4 and Table 2), carvacrol showed a more prolonged effect than thymol (Fig 5E and 5F). The inhibition zone produced by vaporous carvacrol was approximately 0.6 cm, but the zone for vaporous thymol had disappeared by 96 h. The inhibition zone of direct contact thymol decreased at 96 h in a temperature-dependent manner (Fig 5F). Carvacrol was more stable than thymol when the temperature was higher than 80°C.

### Hinokitiol is stable under different pH conditions

**Fig 6:**
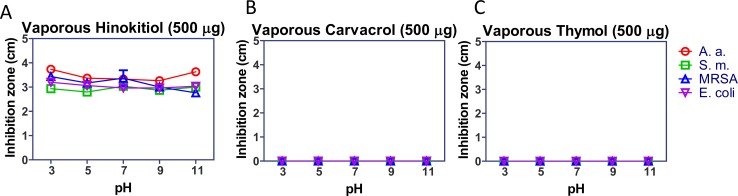
The antibacterial activity of vaporous hinokitiol was stable under different pH conditions. The antibacterial activities of (A) vaporous hinokitiol, (B) vaporous carvacrol, and (C) vaporous thymol were analyzed under different pH conditions.

Most biochemical reactions occur at neutral pH. Environmental pH is a major factor that suppresses microbial colonization [27], but some enteric bacteria produce acid and have high pH resistance [28]. The vapor phase method was used to test the stabilities of hinokitiol, carvacrol, and thymol under various pH conditions to determine whether acidity or alkalinity in the broth agar would interfere with bacterial growth. In the hinokitiol group, the inhibition zones for all microorganisms were similar under the various pH conditions (Fig 6A). The inhibition zone margins of *S*. *mutans*, MRSA, and *E*. *coli* were all cloudy. In the carvacrol and thymol groups, there was no visible inhibition zone under various pH conditions (Fig 6B and 6C), even when the number of inoculated bacteria was increased from 10^6^ to 10^8^ cfu. These results showed that the antibacterial activity of vapor hinokitiol was not affected by pH or the presence of water. The effects of vaporous carvacrol and thymol antibacterial activity were inhibited by water, and the impact of pH on the activity of these compounds could therefore not be verified.

### The phenolic EO compounds exhibit synergistic antibacterial effects

**Fig 7:**
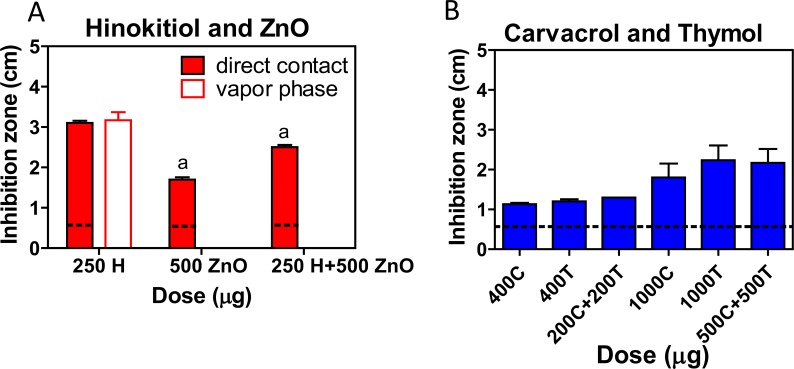
Synergistic antibacterial effects of the phenolic EO compounds. (A) The synergistic effects of hinokitiol (H) and zinc oxide (ZnO) against *A*. *actinomycetemcomitans* (*A*. *a*.) were tested by direct contact and vapor phase agar diffusion tests. (B) The synergism of the anti-MRSA activity of carvacrol (C) and thymol (T) was tested by direct contact agar diffusion tests. a, *P* < 0.01 compared with the direct contact 250H group.

Combination treatment with hinokitiol and ZnO resulted in strong synergistic antibacterial activity and cytotoxicity [29–31]. *A*. *actinomycetemcomitans* was used to study the potential synergistic antibacterial effects of different combinations. The size of the inhibition zones in the direct contact method (from largest to smallest) was 250 μg hinokitiol, followed by 250 μg hinokitiol combined with 500 μg ZnO, then 500 μg ZnO. There was no inhibition zone in the samples treated with 500 μg ZnO, or in the samples treated with 500 μg ZnO combined with 250 μg vaporous hinokitiol as determined by vapor phase method detection (Fig 7A). It has previously been reported that EOs containing carvacrol and thymol can have synergistic effects in combination with antibiotics [32]. The combination of 50% thymol and 50% carvacrol was found to have the highest synergistic antimicrobial activity in another study [33]. However, two different combinations (200 μg carvacrol + 200 μg thymol, and 500 μg carvacrol + 500 μg thymol) showed no synergistic effects in the direct contact method in the present study (Fig 7B).

## Discussion

The strengths of the antibacterial activities for the EOs were hinokitiol > thymol > carvacrol > menthol. The antibacterial working dose and phenotype of carvacrol and thymol were similar, consistent with the findings of Xu et al. [34]. Based on the MIC range, which can be used as a parameter to determine the activity of essential oils [18,35], hinokitiol (MIC = 40–60 μg/mL) had very strong activity, carvacrol and thymol (MIC = 100–400 μg/mL) had strong activities, and menthol (MIC = 500–1000 μg/mL) had relatively moderate activity in this study (Table 2). The kinetics of microbial inactivation depend on the type of microorganism; the type and concentration of biocide; and environmental conditions, such as the temperature, pH, and presence of organic matter [36]. The culture container, shaking rate, air exchange, and visual or ELISA reader interpretation are different between the broth dilution method and kinetic microplate method. These factors can all affect bacterial growth and may lead to different interpretations of the antibacterial activities of the phenolic EO compounds. The microorganism growth and antibacterial activity of the test compounds might be affected by the shaking rate and air exchange during culture. Reducing broth liquid disturbance and air exposure might enhance the antibacterial activity of carvacrol and thymol (Fig 3), but the opposite finding would be expected for menthol. The MIC of menthol was detectable in the broth dilution method (Table 2), although the bacterial growth was not completely inhibited (Fig 4), and the inhibition zone was small or even undetectable (Fig 3).

All microorganisms were sensitive to vapor hinokitiol, regardless of whether they were Gram-positive or Gram-negative. Hinokitiol was previously shown to reduce the microorganisms’ cellular respiration, nucleic acid synthesis, and protein synthesis [37] without damaging the cell membrane or cell wall [23]. The mechanisms by which the phenolic EO compounds exert their antibacterial activity might be correlated with differences in the structures of the cells. The Gram-negative *E*. *coli*. and *A*. *actinomycetemcomitans* were sensitive to vapor carvacrol and thymol, but the Gram-positive *S*. *mutans* and MRSA were not (Fig 6B). The antibacterial effects of carvacrol and thymol were previously attributed to their ability to permeabilize and depolarize the cytoplasmic membrane [34], increasing the levels of reactive oxygen species (ROS) and inducing membrane damage in bacteria [38]. The antibacterial phenotypes of hinokitiol, carvacrol, and thymol were consistent with previous mechanistic studies. However, it is interesting that the antibacterial activities of the EOs towards Gram-positive bacteria in direct contact and for the vapor phase compounds were quite different for carvacrol and thymol. Future detailed physical and biochemical studies are needed to elucidate the mechanisms. Menthol is used more often than other EO compounds in food, oral health products, and dental materials. The mechanism of action of menthol may be related to membrane disruption, leading to cell leakage [18]. However, the antibacterial activity of menthol was the weakest of the four compounds evaluated in this study. These results indicate that the role of menthol in these products may be to induce a fresh and cooling effect instead of antibacterial ability.

The activity of antibiotics might be reduced by heat [39]. Plant-based therapeutics with improved antimicrobial activity and less toxicity are increasingly being accepted as alternatives to conventional antibiotic therapy. The antibacterial activities of hinokitiol, carvacrol, and thymol were stable at various temperatures (Fig 4), and carvacrol was more stable than thymol. The vapor pressures of carvacrol and thymol are 2.96×10^−2^ mmHg and 3.76×10^−2^ mmHg, respectively. The anti-*E*. *coli* activity of thymol gas was previously shown to be strong [40]. In the present study, the antibacterial activity of liquid thymol was slightly decreased when it was assessed at the more than 80°C condition after 96 h, and the antibacterial activity of vaporous thymol was significantly decreased after 96 h (Fig 5F). The relative instability of thymol at high temperatures and its decreased antibacterial activity might have been because the evaporation rate of thymol is faster than that of carvacrol. The antibacterial activity of vaporous hinokitiol was not affected by pH, which was assessed from pH 3 to pH 11, when it was diluted by half with water. However, the antibacterial activities of vaporous carvacrol and vaporous thymol completely disappeared after dilution (Fig 6). These results indicated that hinokitiol is more stable and has higher antibacterial activity at various temperatures in either the liquid or vapor phase, at various pH values, and in different solvents. Dissolving carvacrol and thymol, or the presence of moisture in a hermetic space, might influence their antibacterial efficiency. Modifying these compounds using liposomal and noisome-based diallyl disulfide formulations [24,41] or microcells [42] might improve their solubility, penetration, or bioactivity. Combining the EO with ethyl acetate would also increase EO evaporation to enhance the antibacterial activity and anti-oxidation of vapor phase EO compounds [43]. Using a suitable chemical carrier or combining hinokitiol, carvacrol, and thymol with ethyl acetate might enhance the evaporation and bioactivities of these EO phenolic compounds.

The combination of hinokitiol and ZnO (mass concentration ratio: 1:4, 1:8, 1:32) enhanced the bactericidal activity against clinically isolated *Staphylococci* [30] and showed strong synergistic (mass concentration ratio: 1:2) cytotoxicity [29]. However, combining hinokitiol and ZnO (mass ratio: 1:2) did not cause synergistic antibacterial effects for either liquid or vaporous hinokitiol (Fig 7A). For yeast, there was a synergistic effect only when carvacrol and thymol were used in equal proportions at 100% of the MIC. At 50% of the MIC, no synergistic effect was found for any of the microorganisms [33]. In our study, the MICs of carvacrol and thymol for MRSA were 400 μg/mL and 200 μg/mL, respectively. Treatment with equal mass proportions of 200 μg/mL and 500 μg/mL did not show synergistic effects in the direct contact agar diffusion test. We speculate that this may have been due to the following factors: (1) the working mass concentration ratio was not equal to the working mass ratio, and the synergistic effect disappeared at the incorrect concentration ratio [29]; (2) the ZnO was dissolved in 2.5 N HCl. The hinokitiol can react with strong acid and may have lost its vaporous antibacterial activity. We only confirmed that the hinokitiol was stable from pH 3 to 11 (Fig 6A); (3) ZnO and hinokitiol may combine to form a new product, Zn(hinokitiol)_2_ [44], which may have lost its vaporous antibacterial activity; (4) different methods were used for the analyses. The agar diffusion test may not have been sufficiently sensitive to show the synergistic effects.

Dental patients and dental health-care workers may be exposed to a variety of microorganisms via blood, saliva, and respiratory secretions. In dentistry, besides personal protection, such as eyewear, gloves, gowns, and rubber dams, other considerations, such as a pretreatment mouth rinse and reducing bioaerosols, are vital for infection control in the workplace [45]. The EO of *L*. *gracilis* has significant synergism with several antibiotics. Eugenol has a long history of successful therapeutic use in dentistry, but it can cause allergic reactions in sensitized patients [46]. For patients who are allergic to eugenol, eugenol-free alternatives are available. Carvacrol and thymol showed inhibitory activity against both oral pathogens and food-borne microorganisms [47–49]. The anti-*Candida* activity of carvacrol and thymol were better than that of eugenol, and thymol has previously been used in Orabase [11,50], varnish [51], nano wound dressing [52], and for raw shrimp preservation [53]. Carvacrol was used in apple films [54]. Menthol is widely used in mouth rinse, toothpaste, chewing gum, drinks, and food. However, the antibacterial activity of menthol was relatively weak in this study, but it is often used to modify a food’s flavor, relieve pain, and improve oral malodor.

Hinokitiol has already been used in a mouth cleaning gel [55] and root canal sealer [29]. Liquid and vaporous hinokitiol had the best antibacterial activity, stability, and long-term effects in this study. Hinokitiol exhibits no developmental toxicity [56], no carcinogenic effects [57], no inflammatory response [58], and has low cytotoxicity against normal oral cells [23]. Via *in vitro* genotoxicity testing, carvacrol was shown to have a low genotoxic potential even at a high dose (700 μM), and thymol also did not lead to a genotoxic response [59]. Carvacrol and thymol can bind to the major and minor grooves of B-DNA, but DNA remains in the B-family structure [60]. Hinokitiol, carvacrol, and thymol are safe and have the potential to be applied in dental materials, oral health care products, and food preservation. However, these phenolic EO compounds must be further analyzed in detail prior to their clinical application in dental materials, oral health care products, and for the prevention of food contamination.

## Conclusions

The results of the present study can serve as a guideline for using phenolic EO compounds (hinokitiol, carvacrol, thymol, and menthol) for oral health care products and food preservation. The antibacterial activities of both liquid and vaporous hinokitiol were stable and strong under various temperature and pH conditions. The antibacterial activities of liquid and vapor carvacrol and thymol were also stable at room temperature. The antibacterial activity of thymol was better than that of carvacrol, but the working time and high temperature stability of carvacrol were better than those of thymol. If vaporous carvacrol and vaporous thymol are to be used for antibacterial growth, it is necessary to avoid mixing them with water. Of note, only Gram-negative bacteria were sensitive to vaporous carvacrol and thymol. Menthol had weak antibacterial activity in this study. Continuous agitation decreased the antibacterial effects of menthol but increased those of carvacrol and thymol. The synergistic antibacterial effects of hinokitiol and ZnO, and combinations of carvacrol and thymol, need to be subjected to further analysis in the future. The present antimicrobial and stability data obtained with liquid and vaporous phenolic EO compounds can serve as a guide for the selection of appropriate conditions to be applied in oral health care, food preservation, and infection control in dental hospitals.

## Supporting Information

S1 File The raw data of direct contact agar diffusion tests and vapor phase agar diffusion tests in this study. The raw data include Figs 2(B), 4A, 4B, 4C, 5B, 5D, 5E, 5F, 6A, 6B, 6C, 7A and 7B.
(XLSX)
